# Association Between Time Interval from COVID-19 Vaccination to In Vitro Fertilization and Pregnancy Rate After Fresh Embryo Transfer

**DOI:** 10.1001/jamanetworkopen.2022.36609

**Published:** 2022-10-14

**Authors:** Wenhao Shi, Min Wang, Xia Xue, Na Li, Lijuan Chen, Juanzi Shi

**Affiliations:** 1 Assisted Reproduction Center, Northwest Women’s and Children’s Hospital, Xi'an, China

## Abstract

**Importance:**

There is a lack of information regarding the need to postpone conception after COVID-19 vaccination.

**Objective:**

To investigate the time interval between the first dose of inactivated COVID-19 vaccine and in vitro fertilization (IVF) treatment as well as the rate of pregnancy after a fresh embryo transfer.

**Design, Setting, and Participants:**

This cohort study was conducted at a single public IVF center in China. Female patients aged 20 to 47 years and undergoing IVF treatment were consecutively registered from May 1 to December 22, 2021, with follow-up until March 31, 2022. Patients with SARS-CoV-2 infection before or during IVF treatment and those who underwent 2 or more IVF treatments, received the noninactivated or unknown COVID-19 vaccine, or did not have a fresh embryo transfer were excluded from this study.

**Exposures:**

The vaccinated group (subdivided into 4 subgroups of time interval from first vaccination to fertilization treatment: ≤30 days, 31-60 days, 61-90 days, and ≥91 days) and nonvaccinated group.

**Main Outcomes and Measures:**

Risk ratios (RRs) for the association between the time interval and ongoing pregnancy (pregnancy continued at least 12 weeks).

**Results:**

A total of 3052 female patients (mean [SD] age, 31.45 [3.96] years) undergoing IVF treatment were analyzed in this study. There were 667 vaccinated patients receiving IVF (35 were vaccinated ≤30 days, 58 were vaccinated 31-60 days, 105 were vaccinated 61-90 days, and 469 were vaccinated ≥91 days before fertilization treatment), and 2385 unvaccinated patients receiving treatment. The ovarian stimulation and laboratory parameters were similar among all groups. Ongoing pregnancy was significantly lower in the 30 days or less subgroup (34.3% [12 of 35]; adjusted RR [aRR], 0.61; 95% CI, 0.33-0.91) and the 31 to 60 days’ subgroup (36.2% [21 of 58]; aRR, 0.63; 95% CI, 0.42-0.85). A slightly but not statistically lower rate was found in the 61 to 90 days’ subgroup, and no reduced risk for ongoing pregnancy in the 91 days or more subgroup was observed (56.3% [264 of 469]; aRR, 0.96; 95% CI, 0.88-1.04) compared with the unvaccinated group (60.3% [1439 of 2385], as reference).

**Conclusions and Relevance:**

Findings of this study suggest that receipt of the first inactivated COVID-19 vaccine dose 60 days or less before fertilization treatment is associated with a reduced rate of pregnancy. In patients undergoing IVF treatment with a fresh embryo transfer, the procedure may need to be delayed until at least 61 days after COVID-19 vaccination.

## Key Points

**Question:**

Is there an association between the time of COVID-19 vaccination and rate of pregnancy after in vitro fertilization (IVF)?

**Findings:**

In this cohort study of 3052 female patients undergoing IVF treatment, a significantly reduced pregnancy rate was observed among those who were vaccinated against SARS-CoV-2 30 days or less and 31 to 60 days before IVF treatment. A slightly but not statistically lower rate was found for patients who received vaccination 61 to 90 days before IVF treatment, and no reduced risk for ongoing pregnancy was seen in the 91 days or more subgroup.

**Meaning:**

Findings of this study suggest that IVF treatment with a fresh embryo transfer may need to be delayed until at least 61 days after COVID-19 vaccination.

## Teaser

This cohort study examines the conception risks associated with the timing of the first dose of COVID-19 vaccination among women undergoing in vitro fertilization (IVF) in China.

## Introduction

There are different viewpoints regarding how COVID-19 vaccination affects conception and how long vaccinated couples should wait to conceive. Guidelines, such as those from the American Society for Reproductive Medicine Coronavirus/COVID-19 Task Force^1^ and Centers for Disease Control and Prevention,^2^ recommend that individuals who are planning for pregnancy conceive as soon as possible after receiving the messenger RNA (mRNA) COVID-19 vaccine (at least 3 days before and 3 days after assisted reproduction procedure).

However, the European Society of Human Reproduction and Embryology advises postponing conception for at least a few days after the completion of COVID-19 vaccination (ie, after the second dose) and delaying the start of assisted reproductive technology treatment by up to 2 months.^3^ Delaying pregnancy for 1 month after COVID-19 vaccination is recommended by the Expert Group for Beijing Human Assisted Reproductive Technology Center for Quality Control and Improvement.^4^ Current information on the association of the COVID-19 vaccine with oocytes and sperm, embryo development and implantation, and early stages of pregnancy is lacking. Furthermore, antibody generation against SARS-CoV-2 requires time.

Several preliminary studies found no association between the COVID-19 vaccine and fertility, including ovarian follicular and oocyte performance^5,6^ and embryo development and pregnancy outcomes.^7,8,9^ Despite the clear evidence of immune response (SARS-CoV-2 IgG)^5^ and differences in metabolite levels^10^ in follicular fluid of vaccinated patients undergoing in vitro fertilization (IVF) treatment, the ovarian follicular function was normal. Even with such evidence, individuals receiving assisted reproductive technology treatment remain uncertain about delaying conception and pregnancy after COVID-19 vaccination. To our knowledge, not many reports exist regarding these concerns. In this study, we aimed to investigate the time interval between the first dose of inactivated COVID-19 vaccine and IVF treatment as well as the rate of pregnancy after a fresh embryo transfer.

## Methods

### Study Design and Participants

This observational cohort study registered female patients consecutively at the Northwest Women’s and Children’s Hospital, a public assisted reproductive technology center in China. The study was reviewed and approved by the ethics committee of Northwest Women’s and Children’s Hospital and was conducted according to the principles of the Declaration of Helsinki.^11^ Written informed consent was obtained from all participants. We followed the Strengthening the Reporting of Observational Studies in Epidemiology (STROBE) reporting guideline.

Patients undergoing IVF treatment who received their first dose of the COVID-19 vaccine before fertilization treatment were designated as the vaccinated group. Based on the time interval from the first vaccination to fertilization treatment, the patients were further subdivided into 4 subgroups: 30 days or less, 31 to 60 days, 61 to 90 days, and 91 days or more. Patients undergoing IVF treatment who received no vaccination were designated as the unvaccinated group.

Participants were aged 20 to 47 years and undergoing IVF treatment. They were consecutively registered on the day of oocyte retrieval between May 1 and December 22, 2021 (n = 7934) and were followed up until March 31, 2022. Patient characteristics and information on IVF treatment were extracted from the center’s electronic medical records. Race and ethnicity data were not collected because the local population was mainly (approximately 99.5%) Han Chinese.

Both individuals and couples undergoing IVF treatment were invited to complete the online questionnaire, which included queries on vaccine type, date of manufacture, manufacturer name, and adverse reactions experienced after vaccination. Immunization records were accessed through a personal account on ShaanxiYiMaTong, Alipay, WeChat, and other mobile applications in China. Electronic information on each person was established in China's local health departments, to which individuals reported whether they had a high risk for COVID-19 in the past 14 days and whether they had been vaccinated. Such information was obtained from personal accounts on the social network WeChat and the official (ie, initiated, supported, and operated by the local government department) application ShaanxiYiMaTong. For those patients who did not complete the online questionnaire or had incomplete data, a follow-up telephone call was made to ensure a sufficient questionnaire response rate.

**Figure 1.**
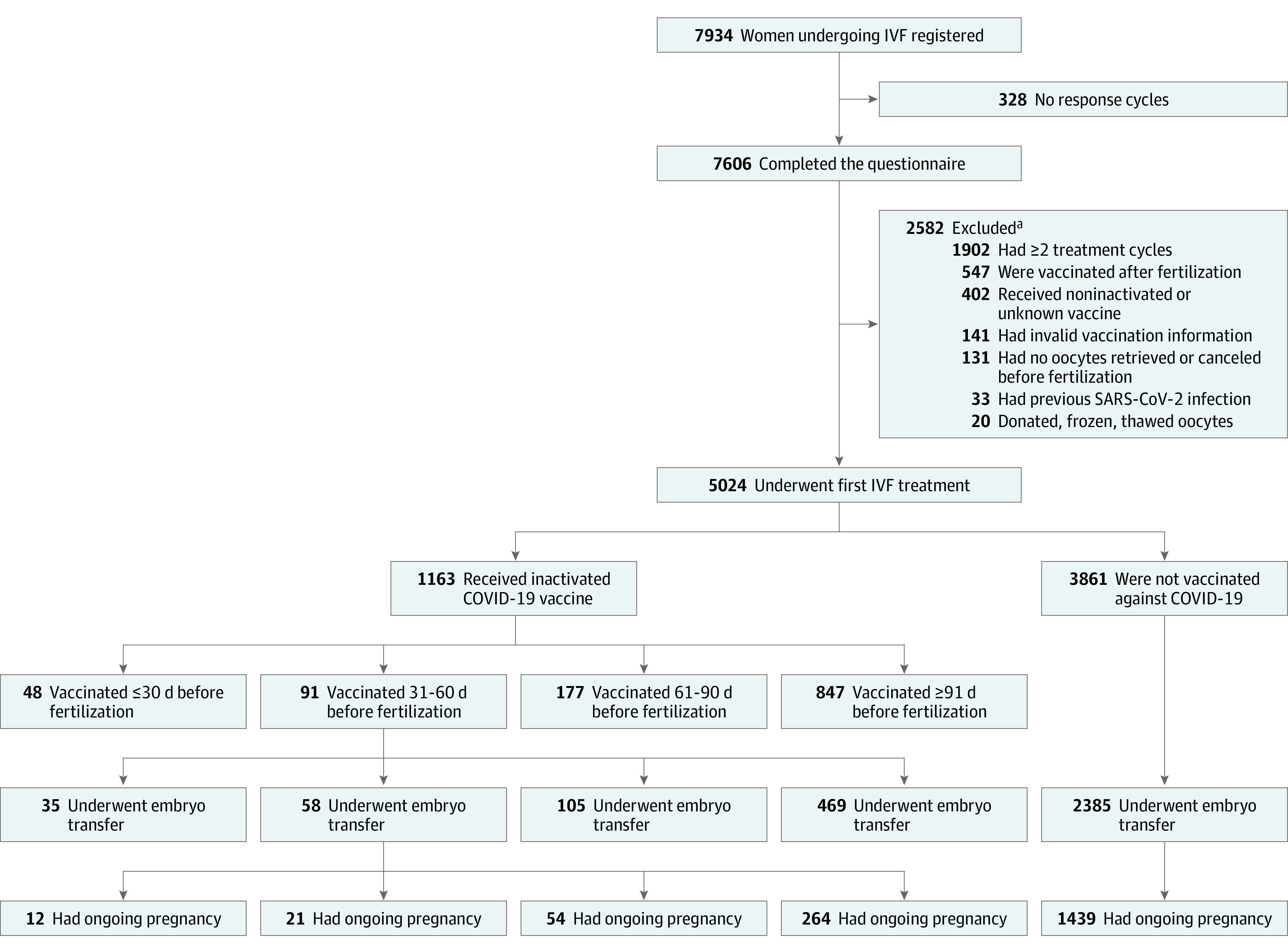
Data Processing IVF indicates in vitro fertilization. ^a^Some patients met more than 1 criterion for exclusion.

The exclusion criteria were as follows: (1) previous SARS-CoV-2 infection; (2) positive polymerase chain reaction test result for SARS-CoV-2 ribonucleic acid (RNA) during IVF treatment; (3) invalid COVID-19 vaccine information; (4) COVID-19 vaccination after fertilization treatment; (5) receipt of donated, frozen, or thawed oocytes; (6) no oocyte retrieval or cycle cancellation before fertilization treatment; (7) loss to follow-up; (8) 2 or more IVF treatment cycles; (9) receipt of noninactivated COVID-19 vaccine or unknown vaccine; and (10) no fresh embryo transfer. A total of 667 patients who received the inactivated COVID-19 vaccine and 2385 patients who were unvaccinated were included in this study (Figure 1).

### IVF Protocols and Laboratory Procedures

Conventional ovarian stimulation for patients with normal or high responses was performed using a standard GnRH (gonadotropin-releasing hormone) agonist protocol (44.7%) or a GnRH antagonist protocol (45.0%). Other protocols were used for patients with a poor ovarian response (10.1%). Detailed ovarian stimulation, trigger, oocyte retrieval, and laboratory procedures were referred to in previous study methods.^12,13,14^ A good-quality, day-3 embryo was defined as having 7 to 10 cells, 15% or less fragmentation, and smooth blastomeres as well as the absence of multinucleation and vacuolation. The expanded blastocysts on day 5 and day 6 were counted using the Gardner grading system.^15^

### Embryo Transfer and Pregnancy Outcomes

Embryo transfer was performed either on day 3 (cleavage stage) or day 5 (blastocyst stage) in the fresh cycle. Day-5 blastocyst transfer was routinely done for patients who had more than 2 good-quality embryos on day 3; otherwise, day-3 embryos were transferred. Approximately 40% of fresh embryo transfers were not performed mainly because of the risk of ovarian hyperstimulation syndrome and asynchrony of the endometrium. Biochemical pregnancy was assessed by a positive serum β-human chorionic gonadotropin level 12 to 14 days after the embryo transfer. Clinical pregnancy was defined as the presence of an intrauterine gestational sac with or without a fetal heartbeat on ultrasonography during the first trimester. Ongoing pregnancy was defined as a clinical pregnancy that continued for at least 12 weeks.

### Statistical Analysis

The statistical analysis was performed using Empower Stats (X&Y Solutions Inc) and R software, version 3.6.1 (R Foundation for Statistical Computing). Continuous variables were presented as median (IQR) and compared by an unpaired, 2-tailed *t* test or 1-way analysis for normally distributed data and a Mann-Whitney test or Kruskal-Wallis test for not normally distributed data. Categorical variables were presented as counts and proportions. Pearson χ^2^ test or Fisher exact probability was used for comparing the categorical variables with the baseline characteristics. A 2-sided *P* < .05 was considered to be statistically significant.

**Figure 2.**
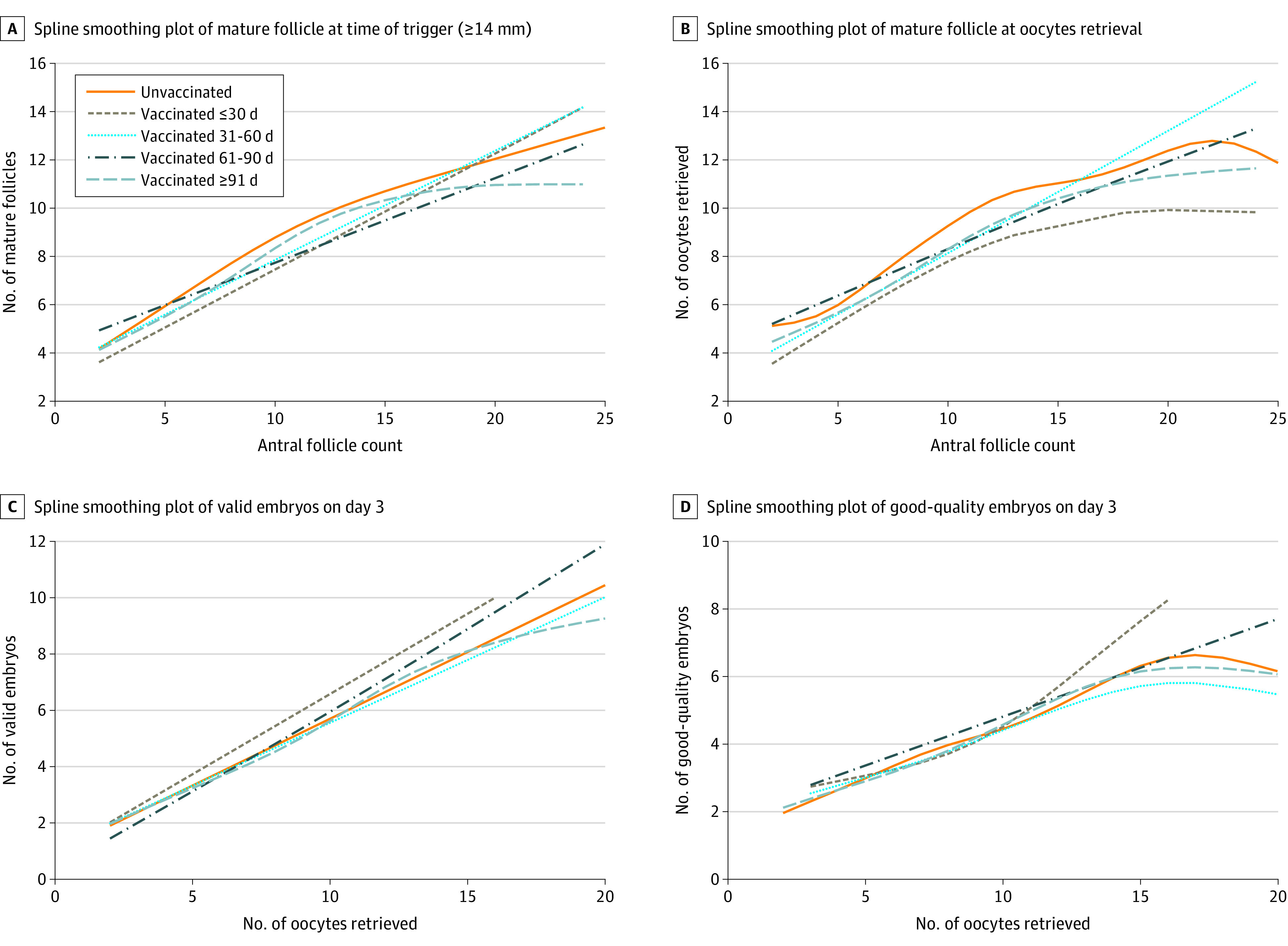
Follicle, Oocyte, and Embryo Performance Data were adjusted for female age (smooth), body mass index, etiological factors of infertility, stimulation protocol, and primary infertility.

**Figure 3.**
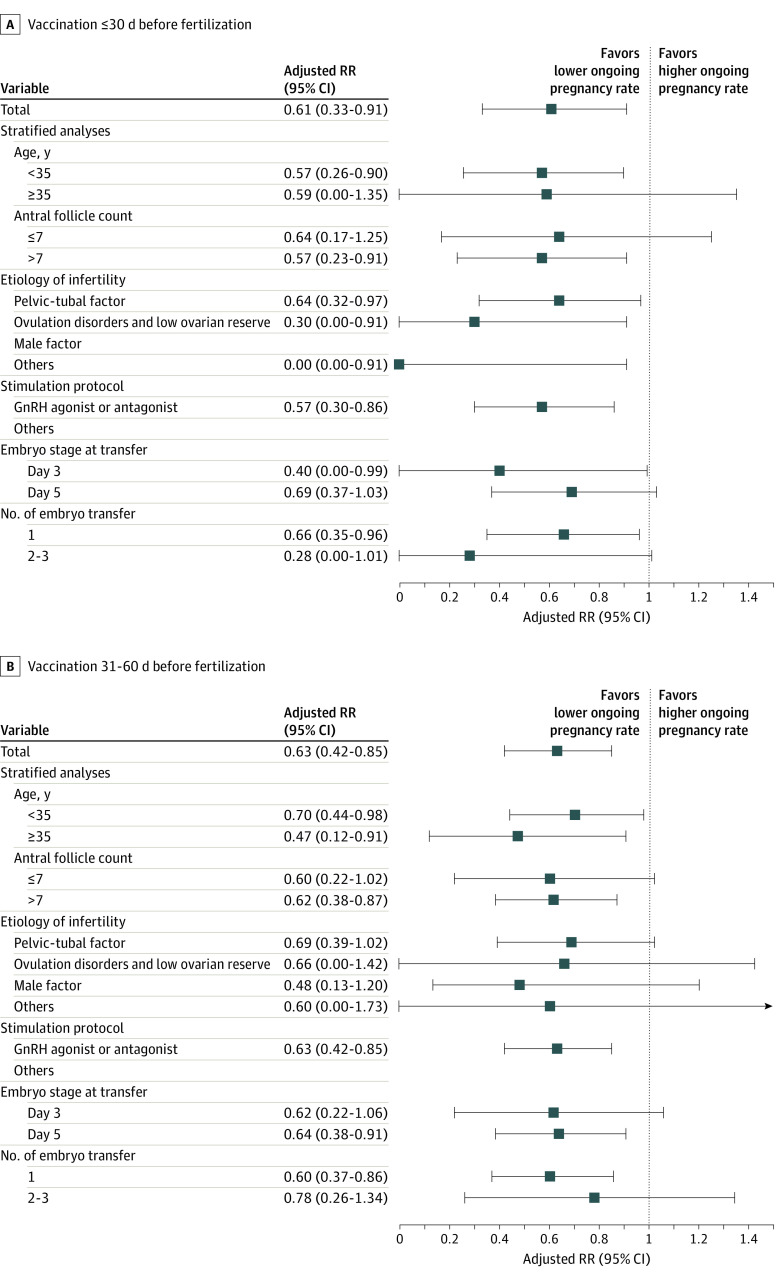
Stratified Analyses of Ongoing Pregnancy Rate With Different Time Intervals Between the First Dose of COVID-19 Vaccine and In Vitro Fertilization (IVF) Treatment Data were adjusted for female age, antral follicle count, etiological factors of infertility, stimulation protocol, embryo stage at transfer, and number of embryo transfers. Unvaccinated cycles were used as a reference (risk ratio [RR] = 1), and an RR less than 1 indicates a reduced ongoing pregnancy rate. GnRH indicates gonadotropin-releasing hormone.

In the generalized additive model, the outcome was the number of mature follicles or oocytes retrieved on day 3, valid embryos on day 3, and good-quality embryos; the explanatory variable was continuous antral follicle count (AFC) or number of oocytes retrieved, which was used to evaluate the follicular or embryo development (Figure 2). The associations between the time interval of COVID-19 vaccination and ongoing pregnancy were assessed using robust Poisson regression, which was assessed using the generalized linear model (Poisson distribution and log link function) (Figure 3 and eFigure in the Supplement). The unadjusted model and adjusted model were established to estimate the risk ratios (RRs) and 95% CIs for the outcomes. The model was adjusted for the potential confounding factors, including female age, AFC, etiological factors of infertility, stimulation protocol, embryo stage at transfer, and number of embryo transfers. Stratified analyses were also performed according to the parameters in the model.

## Results

### Demographic and Baseline Characteristics

A total of 3052 patients undergoing IVF treatment (mean [SD] age, 31.45 [3.96] years) were included in this study. Of these patients, 667 were in the vaccinated (exposed) group and 2385 were in the unvaccinated (unexposed) group. In the vaccinated group, 35 patients were vaccinated 30 days or less before fertilization treatment, 58 patients were vaccinated 31 to 60 days before fertilization treatment, 105 patients were vaccinated 61 to 90 days before fertilization treatment, and 469 patients were vaccinated 91 days or more before fertilization treatment.

The Table details the baseline characteristics of patients and pregnancy outcomes after a fresh embryo transfer. Patients in the vaccinated group were older (median age: 33.0 years for the ≤30 days’ subgroup, 33.0 years for the 31-60 days’ subgroup, 31.0 years for the 61-90 days’ subgroup, and 31.0 years for the ≥91 days’ subgroup vs 31.0 years for the unvaccinated group), had a lower AFC (median: 8.0, 10.0, 11.0, and 11.0 vs 12.0, respectively), and a higher proportion of ovarian factors (20.0% [7 of 35], 10.3% [6 of 58], 16.2% [17 of 105], and 11.3% [53 of 469] vs 11.0% [262 of 2385], respectively) compared with those in the unvaccinated group. There were no differences between the groups in terms of body mass index, stimulation protocol, gravidity, parity, and fertilization method. The adverse effects of vaccination among 667 patients were fever, chills, fatigue, myalgia, and headaches, and they resolved within a few days. All of the effects are listed in eTable in the Supplement.

**Table.** Demographic Characteristics, Time Interval of Vaccination to Fertilization Treatment, and Pregnancy Outcomes

Median (IQR)	Vaccinated group				Unvaccinated group	*P* value
	≤30 d	31-60 d	61-90 d	≥91 d		
Patients undergoing IVF-ET cycles, No.	35	58	105	469	2385	NA
Age, median (IQR), y	33.0 (29.5-36.0)	33.0 (30.0-35.0)	31.0 (29.0-33.0)	31.0 (29.0-35.0)	31.0 (29.0-33.0)	<.001
Age, No. (%)						
<35 y	23 (65.7)	36 (62.1)	86 (81.9)	339 (72.3)	1948 (81.7)	<.001
≥35 y	12 (34.3)	22 (37.9)	19 (18.1)	130 (27.7)	437 (18.3)	
BMI, median (IQR)	23.1 (20.7-25.4)	22.5 (19.9-25.5)	22.5 (20.4-25.2)	22.7 (20.4-25.2)	22.5 (20.3-25.0)	.88
Gravidity, No. (%)						
0	20 (57.1)	29 (50.9)	50 (47.6)	237 (50.5)	1246 (52.4)	.55
1	5 (14.3)	16 (28.1)	26 (24.8)	105 (22.4)	580 (24.4)	
≥2	10 (28.6)	12 (21.1)	29 (27.6)	127 (27.1)	551 (23.2)	
Parity, No. (%)						
0	30 (85.7)	44 (77.2)	83 (79.1)	372 (79.3)	1971 (83.1)	.21
1	5 (14.3)	11 (19.3)	22 (21.0)	83 (17.7)	346 (14.6)	
≥2	0	2 (3.5)	0	14 (3.0)	55 (2.3)	
Etiological factors of infertility, No. (%)						
Pelvic-tubal factor	24 (68.6)	29 (50.0)	68 (64.8)	279 (59.5)	1438 (60.3)	.04
Ovulation disorders and low ovarian reserve	7 (20.0)	6 (10.3)	17 (16.2)	53 (11.3)	262 (11.0)	
Male factor	1 (2.9)	14 (24.1)	17 (16.2)	102 (21.8)	487 (20.4)	
Other factors^a^	3 (8.6)	9 (15.5)	3 (2.9)	35 (7.5)	198 (8.3)	
AFC, median (IQR)	8.0 (6.0-15.5)	10.0 (7.0-13.0)	11.0 (7.0-16.0)	11.0 (7.0-15.0)	12.0 (8.0-16.0)	.003
Stimulation protocol, No. (%)						
GnRH agonist or antagonist	34 (97.1)	58 (100.0)	104 (99.1)	464 (98.9)	2370 (99.4)	.44
Other protocols^b^	1 (2.9)	0	1 (1.0)	5 (1.1)	15 (0.6)	
Mature follicle at trigger (≥14 mm), median (IQR)	8.0 (5.0-11.0)	8.0 (5.0-10.8)	8.0 (5.0-12.0)	8.0 (6.0-11.0)	9.0 (6.0-12.0)	<.001
Fertilization method, No. (%)						
IVF	31 (88.6)	46 (79.3)	91 (86.7)	389 (82.9)	1937 (81.2)	.42
ICSI	4 (11.4)	12 (20.7)	14 (13.3)	80 (17.1)	448 (18.8)	
No. of oocytes retrieved, median (IQR)	7.0 (4.0-10.0)	9.0 (5.0-10.8)	8.0 (6.0-11.0)	8.0 (6.0-11.0)	9.0 (6.0-13.0)	<.001
No. of normal fertilization oocytes (2 pronuclei), median (IQR)	5.0 (3.0-7.0)	6.0 (3.0-7.0)	6.0 (3.0-8.0)	5.0 (3.0-7.0)	6.0 (4.0-8.0)	<.001
No. of valid embryos on day 3, median (IQR)	5.0 (2.5-6.5)	4.0 (2.0-6.0)	5.0 (3.0-7.0)	4.0 (3.0-6.0)	5.0 (3.0-7.0)	<.001
No. of good-quality embryos on day 3, median (IQR)	3.0 (2.0-5.0)	3.0 (1.0-5.0)	3.0 (1.0-5.0)	3.0 (2.0-5.0)	3.0 (2.0-5.0)	.003
Expanded blastocysts on day 5, median (IQR)	0.0 (0.0-3.0)	1.0 (0.0-3.0)	2.0 (0.0-4.0)	2.0 (0.0-4.0)	2.0 (0.0-4.0)	<.001
Total of expanded blastocysts, median (IQR)	2.0 (0.0-4.0)	2.0 (0.0-4.0)	3.0 (0.0-5.0)	2.0 (0.0-4.0)	3.0 (1.0-5.0)	<.001
Embryo transfer, No. (%)						
1	27 (77.1)	46 (79.3)	82 (78.1)	376 (80.2)	1985 (83.2)	.27
≥2	8 (22.9)	12 (20.7)	23 (21.9)	93 (19.8)	400 (16.8)	
Embryo stage at transfer, No. (%)						
Day 3	11 (31.4)	20 (34.5)	34 (32.4)	172 (36.7)	707 (29.6)	.049
Day 5	24 (68.6)	38 (65.5)	71 (67.6)	297 (63.3)	1678 (70.4)	
Biochemical pregnancy, No. (%)	16 (45.7)	31 (53.5)	61 (58.1)	300 (64.0)	1645 (69.1)	<.001
Clinical pregnancy, No. (%)	15 (42.9)	27 (46.6)	59 (56.2)	284 (60.6)	1567 (65.7)	<.001
Ongoing pregnancy, No. (%)	12 (34.3)	21 (36.2)	54 (51.4)	264 (56.3)	1439 (60.3)	<.001

Abbreviations: AFC, antral follicle count; BMI, body mass index (calculated as weight in kilograms divided by height in meters squared); ET, embryo transfer; GnRH, gonadotropin-releasing hormone; ICSI, intracytoplasmic sperm injection; IVF, in vitro fertilization.

^a^ Other factors included endometriosis, uterine infertility and genetic infertility, and unexplained infertility.

^b^ Other protocols included minimal stimulation protocol and progestin-primed ovarian stimulation protocol.

A total of 7606 of 7934 participants completed the online questionnaire, for a response rate of 95.9%. Among the patients who responded to the survey, 1484 (83.7%) received the inactivated vaccine, 8 (0.5%) received the viral vector vaccine, 75 (4.2%) received the protein vaccine, 1 (0.1% received the mRNA vaccine, and 206 (11.6%) were uncertain about the vaccine they received. Of the patients who completed vaccination (2 doses), 531 (79.6%) received the second dose within 2 months after the first dose, whereas 373 (55.9%) were fully vaccinated within 1 month.

### Laboratory and Pregnancy Outcomes

Laboratory and pregnancy outcomes are described in the Table. The number of mature follicles at the time of trigger, which was 14 mm or larger, (median: 8.0 for all subgroups vs 9.0 for the unvaccinated group); number of oocytes (median: 7.0 for the ≤30 days’ subgroup, 9.0 for the 31-60 days’ subgroup, 8.0 for the 61-90 days’ subgroup, and 8.0 for the ≥91 days’ subgroup vs 9.0 for the unvaccinated group); number of normal fertilization oocytes (median: 5.0, 6.0, 6.0, and 5.0 vs 6.0, respectively); and number of valid embryos on day 3 (median: 5.0, 4.0, 5.0, and 4.0 vs 5.0, respectively) were similar in all of the subgroups. Given that these indicators were associated with the basal AFC, the number of mature follicles per AFC and the number of oocytes retrieved per AFC were generally used to evaluate follicular development and oocyte recovery efficiency.

Similarly, embryo development was generally evaluated by the number of day-3 valid embryos per oocyte and the number of good-quality day-3 embryos per oocyte. The number of mature follicles at the time of trigger per AFC (median: 0.7 for the ≤30 days’ subgroup, 0.8 for the 31-60 days’ subgroup, 0.8 for the 61-90 days’ subgroup, and 0.8 for the ≥91 days’ subgroup vs 0.8 for the unvaccinated group), number of oocytes retrieved per AFC (median: 0.8 for all subgroups and unvaccinated group), number of day-3 valid embryos per oocytes retrieved (median: 0.7 for the ≤30 days’ subgroup and 0.6 for the rest of the subgroups and unvaccinated group), and number of good-quality day-3 embryos per oocytes retrieved (median: 0.4 for all subgroups and unvaccinated group) were similar in all subgroups (Figure 2).

**Figure 4.**
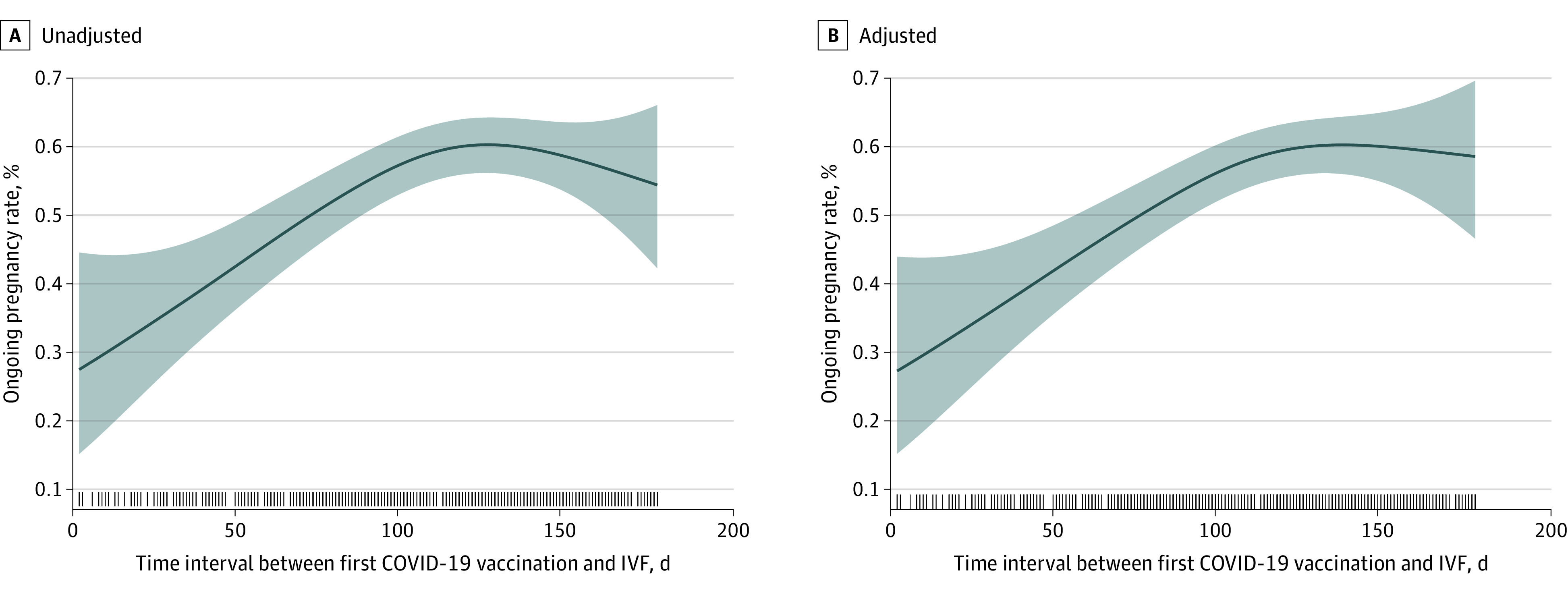
Ongoing Pregnancy Rate by Time Interval of Vaccination to Fertilization Treatment as a Continuous Variable The horizontal axis represents the time interval between vaccination and in vitro fertilization (days, as a continuous variable), and the vertical axis represents the ongoing pregnancy rate. Spline smoothing plot represents the ongoing pregnancy rate according to the time interval of vaccination to fertilization treatment. The model was adjusted for female age (smooth), antral follicle count, etiological factors of infertility, stimulation protocol, embryo stage at transfer, and number of embryo transfers. The shaded areas represent the 95% CIs, and the marks along the x axis represent the distribution of participants in the study.

There were 667 vaccinated and 2385 unvaccinated patients who underwent a fresh embryo transfer. The rate of ongoing pregnancy increased with the time interval in each vaccination subgroup and the unvaccinated group (34.3%, 36.2%, 51.4%, and 56.3%, respectively, and 60.3% in the unvaccinated group) (Table). The biochemical pregnancy rate and clinical pregnancy rate also had a similar pattern. The adjusted RRs for ongoing pregnancy were significantly lower in the 30 days or less vaccinated subgroup (adjusted RR, 0.61; 95% CI, 0.33-0.91) and the 31 to 60 days’ subgroup (adjusted RR, 0.63; 95% CI, 0.42-0.85) (Figure 3). However, no significantly lower adjusted RRs were observed in the 61 to 90 days’ subgroup and the 91 days or more subgroup (adjusted RR, 0.96; 95% CI, 0.88-1.04) (eFigure in the Supplement) compared with the unvaccinated patients (reference). Furthermore, the adjusted RRs of the ongoing pregnancy rate between the vaccinated subgroups were increased with increasing time intervals from first vaccination to fertilization treatment in both total and stratified analyses (Figure 3 and eFigure in the Supplement). To further clarify this pattern, the performance of the ongoing pregnancy rate was analyzed according to time intervals as a continuous variable. The ongoing pregnancy rate was the lowest when the time interval (days) was 0, and then the rate linearly increased with the time interval until approximately 90 days to a platform (Figure 4).

## Discussion

A significantly reduced pregnancy rate was observed among patients who received the first dose of COVID-19 vaccine 30 days or less or 31 to 60 days before IVF treatment. A slightly lower but not statistically different rate was found in those who received COVID-19 vaccination 61 to 90 days before fertilization treatment, and no reduced risk for ongoing pregnancy was found in the 91 days or more subgroup. Furthermore, this risk decreased with increasing time intervals between COVID-19 vaccination and IVF treatment.

Until now, only a few studies have examined the association of the COVID-19 vaccine with infertility in individuals undergoing IVF treatment. Orvieto et al^16^ reported no differences in ovarian stimulation and embryologic variables observed in 36 couples who resumed IVF treatment 7 to 85 days after receiving the mRNA COVID-19 vaccine. Moreover, Aharon et al^17^ reported no adverse implications for stimulation or early pregnancy outcomes after IVF treatment in a comparison of 222 women who received the mRNA vaccine with 983 women who were unvaccinated. Odeh-Natour et al^18^ examined the implications of the Pfizer-BioNTech mRNA vaccine for IVF treatment, oocyte and embryo quality, and pregnancy outcomes in 37 women and found no association between the vaccine and IVF treatment outcomes or ovarian reserves in the subsequent IVF treatment cycle. Avraham et al^8^ also reported no difference in ovarian response or pregnancy rates between the vaccinated and unvaccinated groups of 200 patients, 14 to 68 days after COVID-19 vaccination, but the investigators did not mention the time interval from vaccination to fertilization treatment. Aizer et al^9^ reported no difference in implantation with their subsequent frozen-thawed embryo transfer among 141 patients (after SARS-CoV-2 infection for 44 patients and after COVID-19 vaccination for 220 patients). In addition, Huang et al^19^ found no implication for the number of oocytes retrieved, good-quality embryo rate, and clinical pregnancy rate in 146 individuals who were vaccinated with an inactivated-virus vaccine. Furthermore, in Huang et al,^19^ no significant differences were found in either laboratory or pregnancy outcomes based on the time interval from the COVID-19 vaccination to IVF cycle initiation (≤1 month, >1–2 months, and >2 months). Dong et al^7^ also found that the COVID-19 vaccination status of both partners in infertile couples, different types of vaccines, and time intervals from vaccination to fertilization treatment were not associated with embryo quality and pregnancy rates in 735 infertile couples undergoing IVF treatment.

In the present study, the time interval was defined as the time from complete COVID-19 vaccination to the first embryo transfer and was compared among patients with an interval of 30 days or less, 31 to 60 days, 61 to 90 days, and 91 days or more. However, in the previous studies, only the patients who completed 2 doses of the mRNA or inactivated vaccine before the onset of controlled ovarian hyperstimulation were included in the vaccinated group. This inclusion was inconsistent with the definition in this study, which included patients undergoing IVF treatment who received their first dose of the vaccine before fertilization treatment in the vaccinated group. Thus, the present study analyzed more patients who received IVF treatment immediately after vaccination. This variation in selecting participants may have been 1 of the factors in the inconsistencies in the outcome between the present and previous studies.

Only those patients who received the inactivated COVID-19 vaccine (83.7% of patients) were compared with the unvaccinated group in the present study; therefore, the interpretation of the results should be cautious. Because of the small number of patients with other types of vaccinations (from 0.1% receiving mRNA vaccines to 4.2% receiving protein vaccines), we excluded patients who were vaccinated with anything other than inactivated vaccines. Different types of vaccines may change IVF performance, and COVID-19 vaccines vary among countries. In the US and Europe, most vaccines used were mRNA vaccines. In China and other Asian countries, the main vaccines used were inactivated vaccines.

In this study, 667 patients received 2 vaccine doses. The second dose was administered 4 to 7 weeks after the first dose. Furthermore, 79.5% of patients received the second vaccine dose within 2 months after the first dose, whereas 55.8% of patients completed vaccination within 1 month. The ovarian stimulation protocol lasted for 2 to 3 weeks before fertilization treatment. Hence, some of the participants who were vaccinated 30 days or less before IVF treatment had the second dose during the time of embryo implantation. Vaccination during implantation may have been associated with a low pregnancy rate. The adverse effects of the vaccine may have been associated with early pregnancy outcomes. However, none of the 35 patients in the 30 days or less subgroup had adverse effects (eTable in the Supplement); therefore, the low pregnancy rate in patients who were vaccinated 30 days or less before IVF treatment might not have been associated with the adverse effects of vaccination. Nevertheless, the potential inflammatory changes after vaccination may have interfered with the processes of early embryonic development^20^ and subsequent implantation, similar to SARS-CoV-2 infection.^21^

Other variables, such as genetic and environmental factors, might have differed in patients with a long duration since the vaccination. Based on vaccination policies in different regions, essential workers were the first to receive the vaccines and others were vaccinated subsequently. There was a possibility that the first vaccinated population was healthier than the general population, which may have created potential selection bias in selecting participants for this study. Personal information, such as occupation, and priorities of the vaccinated population should have been collected, but future studies need to address this issue.

Furthermore, the appearance of SARS-CoV-2 variants or strains may have altered the interpretation of the results, including the subsequent update of infection detection and vaccination activity. The current vaccines may not have provided the same degree of protection against evolving variants^22^; therefore, continuing with regular boosters has become the norm for continued protection. Many studies are needed to clarify the association between the time interval from COVID-19 vaccination to fertilization treatment and pregnancy outcomes.

### Limitations

Several limitations must be considered when interpreting the findings of this study. Only the participants who received inactivated vaccines were analyzed, and therefore, the conclusion should be cautiously interpreted for other types of vaccines. Only the pregnancy outcome of a fresh embryo transfer was analyzed; the outcome of a frozen-thawed embryo transfer was beyond the scope of this study. Furthermore, live birth data were unavailable owing to the short follow-up period. Future studies have been planned to follow-up on the frozen-thawed embryo transfers and their offspring. Other variables, such as race and ethnicity, occupation, and priority of the vaccinated population, may be associated with potential selection bias. Because of the observational nature of this study, the implications of vaccination received less than 1 month before fertilization treatment for early pregnancy outcomes needs to be further substantiated by additional scientific studies.

## Conclusions

In this cohort study, receipt of the first inactived COVID-19 vaccine dose 60 days or less before IVF treatment was associated with a reduced rate of pregnancy. For patients undergoing IVF treatment with a fresh embryo transfer, the procedure may need to be delayed until at least 61 days after COVID-19 vaccination.

## Notes

Supplement. **eTable.** The Side Effects of Vaccination (After the First or Second Dose, or Both) **eFigure.** Stratified Analyses of Ongoing Pregnancy Rate With Different Time Intervals Between the First Dose of COVID-19 Vaccine and In Vitro Fertilization
