# Supplementary material for: Association Between Time Interval from COVID-19 Vaccination to In Vitro Fertilization and Pregnancy Rate After Fresh Embryo Transfer
Source: JAMA Netw Open. 2022 Oct 14;5(10):e2236609. doi: 10.1001/jamanetworkopen.2022.36609 (PMC9568801; doi:10.1001/jamanetworkopen.2022.36609)
Supplement: Supplement. — eTable. The Side Effects of Vaccination (After the First or Second Dose, or Both) eFigure. Stratified Analyses of Ongoing Pregnancy Rate With Different Time Intervals Between the First Dose of COVID-19 Vaccine and In Vitro Fertilization [file jamanetwopen-e2236609-s001.pdf]

Supplemental Online Content

Shi W, Wang M, Xue X, Li N, Chen L, Shi J. Association between time interval from COVID-19 vaccination to in vitro fertilization and pregnancy rate after fresh embryo transfer. *JAMA Netw Open*. 2022;5(10):e2236609. doi:10.1001/jamanetworkopen.2022.36609

eTable. The Side Effects of Vaccination (After the First or Second Dose, or Both)

eFigure. Stratified Analyses of Ongoing Pregnancy Rate With Different Time Intervals Between the First Dose of COVID-19 Vaccine and In Vitro Fertilization

This supplemental material has been provided by the authors to give readers additional information about their work.

© 2022 Shi W et al. *JAMA Network Open.*

eTable. The Side Effects of Vaccination (After the First or Second Dose, or Both)

	Vaccinated group			
	≤ 30 days	31-60 days	61-90 days	≥91 days
IVF-ET cycles, n	35	58	105	469
Total side effects, n	0	7	3	13
Pain at injection site, n		2	1	4
Menstrual disorder, n		2	-	1
Flu-like symptoms, n		3	2	7
Redness, swelling, itching and rash at the injection site, n		-	-	1

© 2022 Shi W et al. *JAMA Network Open.*

eFigure. Stratified Analyses of Ongoing Pregnancy Rate With Different Time Intervals Between the First Dose of COVID-19 Vaccine and In Vitro Fertilization

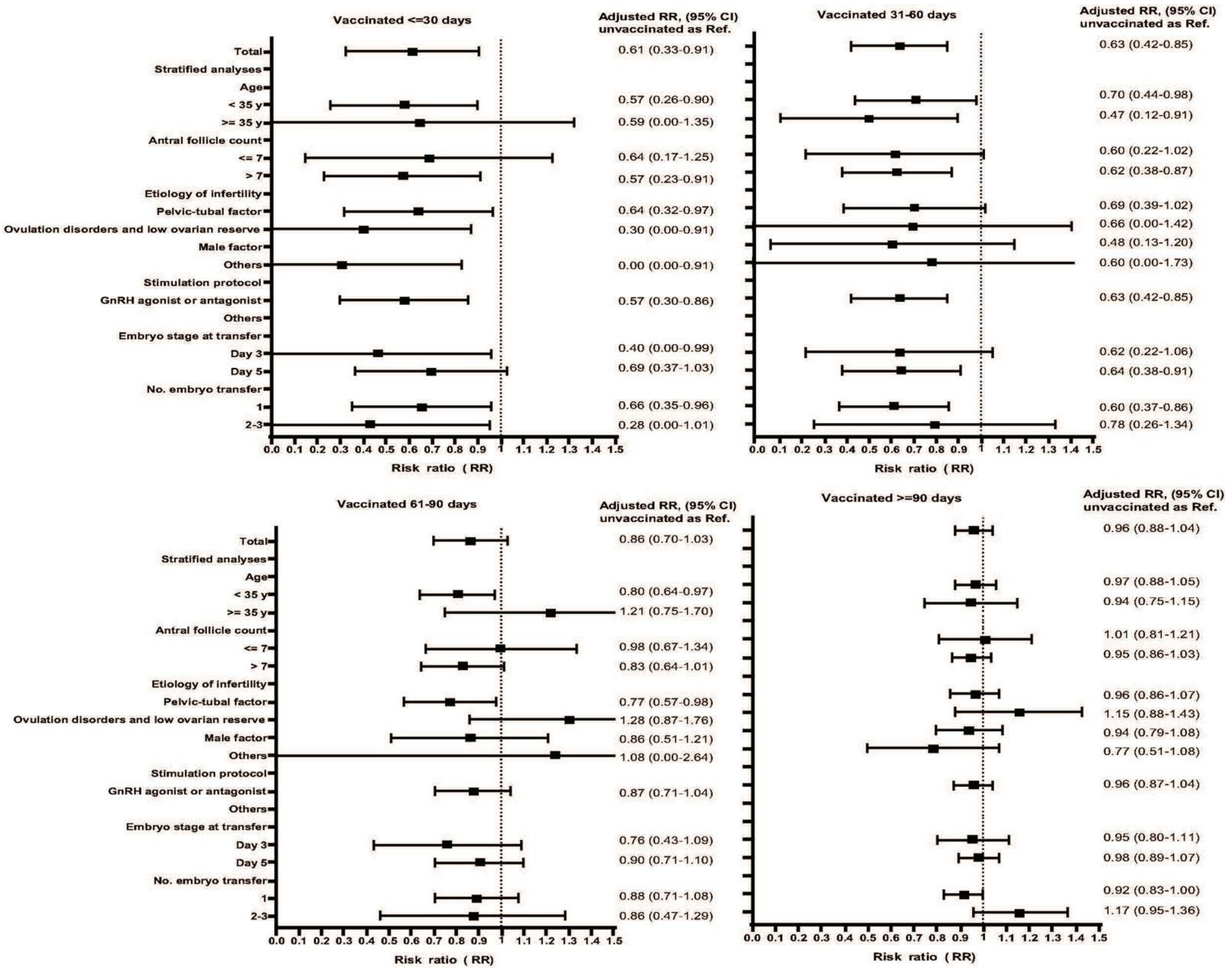

© 2022 Shi W et al. *JAMA Network Open.*
